# Supplementary material for: Saccharomyces boulardii (CNCM I-745) improves intestinal damage in sepsis by remodeling intestinal flora structure
Source: Front Cell Infect Microbiol. 2025 Jul 31;15:1602792. doi: 10.3389/fcimb.2025.1602792 (PMC12350329; doi:10.3389/fcimb.2025.1602792)
Supplement: Supplementary file 1 [file DataSheet1.zip › summary/summary/7_Differential_analysis/1_diff_abundance/7_Species/Group.DW4_vs_DW0_vs_CW4_vs_CW0_vs_BW4_vs_BW0_vs_AW4_vs_AW0/Group.DW4_vs_DW0_vs_CW4_vs_CW0_vs_BW4_vs_BW0_vs_AW4_vs_AW0_diff_barplot.pdf]

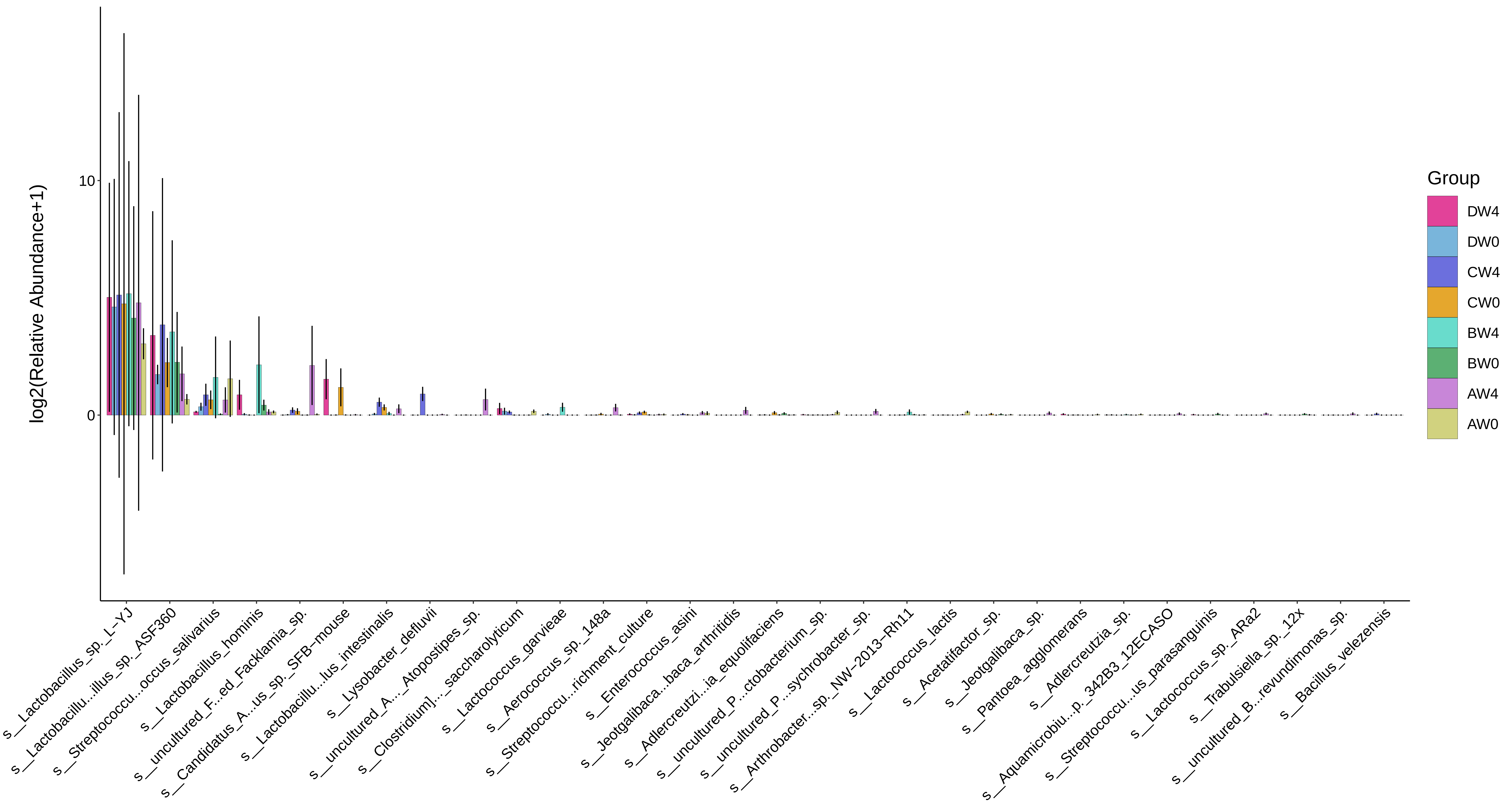

log2(Relative Abundance+1)
10
0
s__Lactobacillus_sp._L−YJ
s__Lactobacillu...illus_sp._ASF360
s__Streptococcu...occus_salivarius
s__Lactobacillus_hominis
s__uncultured_F...ed_Facklamia_sp.
s__Candidatus_A...us_sp._SFB−mouse
s__Lactobacillu...lus_intestinalis
s__Lysobacter_defluvii
s__uncultured_A..._Atopostipes_sp.
s__Clostridium]...saccharolyticum
s__Lactococcus_garvieae
s__Aerococcus_sp._148a
s__Streptococcu...richment_culture
s__Enterococcus_asini
s__Jeotgalibaca...baca_arthritidis
s__Adlercreutzi...ia_equolifaciens
s__uncultured_P...ctobacterium_sp.
s__uncultured_P...sychrobacter_sp.
s__Arthrobacter...sp._NW−2013−Rh11
s__Lactococcus_lactis
s__Acetatifactor_sp.
s__Jeotgalibaca_sp.
s__Pantoea_agglomerans
s__Adlercreutzia_sp.
s__Aquamicrobiu...p._342B3_12ECASO
s__Streptococcu...us_parasanguinis
s__Lactococcus_sp._ARa2
s__Trabulsiella_sp._12x
s__uncultured_B...revundimonas_sp.
s__Bacillus_velezensis
Group
DW4
DW0
CW4
CW0
BW4
BW0
AW4
AW0
